# Characteristics of Patients Hospitalized with 2009 H1N1 Influenza in a Tertiary Care Hospital in Southern Saudi Arabia

**DOI:** 10.4084/MJHID.2012.002

**Published:** 2012-01-06

**Authors:** Adnan Agha, Abdulqader Alrawi, Cesar V. Munayco, Mohammed S. AlAyed, Mohammad Al-Hakami, Hassan Korairi, Abdelhaleem Bella

**Affiliations:** 1 Department of Internal Medicine, Armed Forces Hospital Southern Region, Khamis Mushyt, Saudi Arabia; 2 Department of Preventive Medicine, Armed Forces Hospital Southern Region, Khamis Mushyt, Saudi Arabia; 3 Dirección General de Epidemiología, Ministry of Health of Peru; 4 Department of Pediatrics, College Of Medicine, Najran University, Najran, Saudi Arabia; 5 Department of Internal Medicine, University of Dammam, Saudi Arabia

## Abstract

**Background:**

Pandemic influenza A (H1N1) virus emerged and spread globally in the spring of 2009. We describe the clinical features of the patients who were hospitalized with 2009 H1N1 influenza July 2009 to June 2010 in a tertiary care hospital in Khamis Mushyt, Saudi Arabia. We analyzed the clinical and laboratory variables in order to determine predictors of poor outcome.

**Methods:**

We performed a prospective study in all patients who were hospitalized for at least 48 hours and with a positive test for 2009 H1N1 virus through RT-PCR(real time polymerase chain reaction). Their epidemiological, clinical, biochemical characteristics were collected and the hospital course of the patients with eventual outcome (discharge or death) was observed. We applied a logistic regression analysis to determine the best predictor of death.

**Results:**

A total of 52 patients (15 males) were adults and 65 were pediatrics (< 12 years of age) (19 males). The common presenting signs and/or symptoms associated with the disease was fever >38.5 ºC (n=85; 72.6%), dry cough (n=81; 69.2%), dyspnea (n=40; 34.5%), tachycardia (n=96; 83.5%) and saturation less than 90% in room air on pulse oximetry (n=65; 55.6%). The complications included pneumonia (40.2 %), intensive care unit admission (19.2%) and death (16.7%).

**Conclusions:**

We found that hypoxia at admission was the most important predictive factor of poor outcome (death) with area under curve of 0.768.

## Introduction

During the spring of 2009, a novel influenza A (H1N1) virus of swine origin caused human infection and acute respiratory illness in Mexico.^1^, ^2^ After initially spreading across the United States and Canada,^3^ the virus spread globally, resulting in the first influenza pandemic since 1968 with circulation outside the usual influenza season in the Northern Hemisphere.^4^ As of March 2010, almost all countries had reported cases, and more than 17,700 deaths among laboratory-confirmed cases had been reported to the World Health Organization (WHO), which significantly underestimates the pandemic’s impact.^5^ In the United States alone, an estimated 59 million illnesses, 265,000 hospitalizations, and 12,000 deaths had been caused by the 2009 H1N1 virus as of February 2010, while in April 2010 WHO announced that H1N1 had entered a post-pandemic phase.^6^

The original Pandemic 2009 H1N1 virus genome actually derives six genes from triple-reassortant North American swine virus lineages and two genes from Eurasian swine virus lineages.^7^ The first case of pandemic influenza A (H1N1) virus was reported in Saudi Arabia on June 3, 2009.^8^ On September 12, 2009, the Saudi Ministry of Health issued a national plan for the management of the flu-like pandemics, specifically pandemic influenza A (H1N1) virus infections.^9^ Although there have been various risk factors identified in recent literature from Saudi Arabia for poor outcome among patient with 2009 H1N1 infection, like delay in initiation of antiviral therapy, young age female sex and patients with chest pain, confusion or loss of consciousness;^10^ but to-date there has been no study done to identify a model that could predict risk factor for poor outcome in these patients.

Here we attempt to describe the clinical and biochemical features of the patients who were hospitalized with 2009 H1N1 influenza July 2009 to June 2010 in a tertiary care hospital in Khamis Mushyt, in the southern region of Saudi Arabia and the hospital course of these patients outcome and the predictive factors associated with poor outcome.

## Patients and Methods

### Study design

We performed a prospective study of all patients who were admitted to Internal Medicine or Pediatric Department, at Armed Forces Hospital Southern Region Khamis Mushyt From July 3, 2009, to June 1, 2010.

### Selection of patients

We review all the records of hospitalized patients that had at least 48 hours with influenza-like illness (temperature of 37.8°C or higher or cough or sore throat) and had 2009 H1N1 virus infection confirmed with RT-PCR at National Health Virology Laboratory in Riyadh. The RT-PCR assay was performed using kits of the Centers for Disease Control and Prevention (RT-PCR protocol for detection and characterization of swine influenza, version 2009, CDC REF.#I-007-05).^11^

We identified patients prospectively through daily reports regarding case-level information (including hospitalization status) from departmental reports and authors personally collected clinical information for each hospitalized patient after informed consent.

We gathered data from the hospitalized patients through standardized sheet record that included demographic data, influenza-vaccination history for the previous year, underlying medical conditions, clinical signs and symptoms, laboratory tests, radiographic findings, treatment course, requirement for ventilation or intensive care and eventual outcome. Specimens for bacterial infections were sent for all patients to rule out secondary infections. For time calculations, the day of admission was considered to be hospital day 0. The body-mass index (BMI, the weight in kilograms divided by the square of the height in meters) was calculated, for patients for whom height and weight were available, to determine whether the patient was obese (with obesity defined as a BMI of 29.9 or higher in adults 13 years of age or older or a BMI percentile of 95 to 100 in children between the ages of 2 and 12 years) or morbidly obese (BMI ≥39.9 in adults only); the BMI was calculated in pregnant women based on their first antenatal visit in medical charts. Fever was defined as documented temperature of ≥37.5°C with high grade temperature being defined as documented temperature of ≥38.5°C. Complicated pandemic influenza was defined in the presence of radiologically confirmed pneumonia, septic shock, multiorgan failure, central nervous system (CNS) involvement, etc. or secondary bacterial infections of the upper respiratory tract defined and diagnosed by standard methods. A severe case was defined as requiring admission to an intensive care unit (ICU) or death. We performed separate analysis for patients who either died or were admitted to an ICU. We did a multivariate logistic-regression analysis to further investigate associations with the severity of illness.

## Results

### Clinical features

A total of 52 patients (15 males) were adults and 65 were pediatrics (19 males). There were 15 patients in the age group 60 years and above, 35 patients in the age group 21–60 years, 18 patients in the age group 6–20 years and 41 patients in the age group 0–5 years (see table 1). The common presenting symptom associated with the disease was fever >38.5 ºC present in 85 cases (72.6%), dry cough 81 cases (69.2%), dyspnea 40 cases (34.5%), vomiting 40 cases (34.2%) and diarrhea 45 cases (38.8%). The most significant signs associated with pandemic 2009 H1N1 infection in our hospitalized patients were tachycardia present in 96 cases (83.5%), room air desaturation on pulse oximetry less than 95% present in 65 cases (55.6%), tachypnea more than 20 breaths per minute present in 82 cases (97.6%), and crackles on chest auscultation present in 57 cases (49.1%) (see table 1 for details).

### Co-morbidities

There were 10 patients who were pregnant (10.5%) and the common co-morbidities among the all patients hospitalized with H1N1 pandemic were chronic kidney disease present in 5 cases (4.3 %), Sickle cell anemia present in 8 cases (6.9%), Congenital cardiovascular disease present in 5 cases (4.3%), Asthma present in 7 cases (6.8%) and Epilepsy present in 11 cases (9.5%).

### Radiological and Laboratory Findings

Most of the patient hospitalized with 2009 H1N1 infection had right sided infiltrates on chest x-ray in 24 cases (20.5%) and normal chest x-ray in 28 patients (41.0%).

Arterial blood gases showed hypoxia in 47 patients (56.6%), while blood work showed anemia in 36 patients (30.8%), normal white blood count in 82 cases (70.1%), low white blood count in 11 patients (9.4%), raised alanine transaminase in 19 patients (16.8%) *Morbidity:* The patients hospitalized with 2009 H1N1 infection had complications like requiring non-invasive ventilation in 4 patient (3.4%) and invasive mechanical ventilation in 16 cases (13.7%). The mortality in our patients was 18 cases (16.7%).

### Pregnancy

A total of 10 patients who were pregnant (10.5 %), there were 0 deaths among pregnant patients and the mortality was slightly better when compared to the total population mortality of 16.7%.

### Predictive Model

We tried to identify the factors predicting the poor outcome in the patient hospitalized with 2009 H1N1 infection. The important factors associated with morbidity and mortality were need for non-invasive mechanical ventilation (n=4), invasive mechanical ventilation (n=16), type 2 respiratory failure (n=7), bilateral chest infiltrates (n=11) and low white blood cell count (n=11) but the small number of patients did not allow to do more stratified analysis. The logistic regression analysis showed that the hypoxia at admission is the most important variable (n=65) after adjusted by duration of symptoms, age and sex, the model has an area under the curve being 0.768.

**Figure 1:**
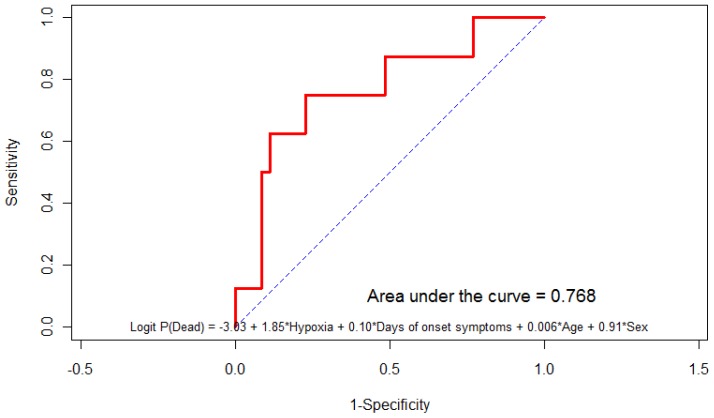
Predictive model for predicting poor outcome in patients hospitalized with 2009 h1n1 pandemic

Predictive Model=Log P (dead)-3.03+1.85* Hypoxia +0.10 * Days of onset of symptoms +0.006 * Age+0.91*sex (see figure 1).

## Discussion

In this study, we have shown that pandemic influenza caused considerable morbidity in a significant proportion of hospitalized adults and that the use of antiviral drugs was beneficial in hospitalized patients. The distribution of influenza A (H1N1) cases by age was similar to the distribution of cases observed worldwide, suggesting the possibility of varying levels of immunity in the young age group although more than 90% of influenza-related deaths occur in patients in the older age group.^12^ Underlying medical conditions that have been reported in patients worldwide who were hospitalized with seasonal influenza have included diabetes and cardiovascular, neurologic, and pulmonary diseases, including asthma.^13^ Frequently reported complications have included pneumonia, bacterial coinfection, and exacerbation of underlying medical conditions, such as congestive heart failure.^14^

Our study summarizes the clinical findings regarding patients who were hospitalized for the treatment of 2009 H1N1 influenza in pandemic period over one year. Studies have shown that early therapy with oseltamivir in patients with 2009 H1N1 virus infection may reduce the duration of hospitalization and the risk of progression to severe disease requiring ICU admission or resulting in death.^15^,^16^,^17^ Studies have also shown that patients with immunosuppressant states such as cancer and/or stem cell transplantation who develop 2009 H1N1 have not been associated with more poor outcome.^18^ International studies have shown that In-hospital mortality was higher in patients with pneumonia than in the others (5.2% vs. 0%; p < 0.001) while the absence of comorbid conditions (odds ratio [OR], 2.07; 95% confidence interval [CI], 1.32–3.24) was found to be an independent risk factor for pneumonia, whereas early (≤48 h) oseltamivir therapy (OR, 0.29; 95% CI, 0.19–0.46) was a protective factor.^19^

When compared to the local studies in Saudi Arabia, risk factor associated with severe 2009 H1N1 infection leading to either complications or intensive care admission have been delay in initiation of antiviral therapy, history of opium inhalation, younger age group and in female dependents and certain symptoms like; productive cough, hemoptysis, chest pain, confusion, loss of consciousness.^20^, ^21^

In our study involving 117 patients with 2009 H1N1 virus 40.2% had pneumonia on chest x-ray, 19.2% required mechanical ventilation and 16.7% died; all patients received oseltamivir. The factor most predictive of morbidity and mortality was hypoxia at admission that had an area under curve of 0.768 with OR for hypoxia being 6.356 with 95% CI being 1.16–34.55. Although the number of patients in this study was small and it was single centered experience, we may identify patient at high risk for morbidity and mortality using this model.

## Conclusion

During the evaluation period, 2009 H1N1 influenza caused severe illness including pneumonia (40.2 %), intensive care unit admission (19.2%) and death (16.7%) in single tertiary care hospital in the southern region of Saudi Arabia over a one year period. The most significant factor in our small study that was predictive of morbidity and mortality in our patients was hypoxia at admission that had an area under curve of 0.768 (see figure 1). Identifying the patients with hypoxia in suspected cases of 2009 H1N1 influenza infection is therefore prudent as these patients may be at greater risk for morbidity and mortality. Although 2009 H1N1 infection is now considered to be in post-pandemic phase with low virological activity, identifying risk factors such as hypoxia on presentation in these patients may still be necessary to optimize treatment strategies and therefore further studies to needed identify/confirm the factors predicting poor outcomes in patients admitted with 2009 H1N1 infection.

## Figures and Tables

**Table 1:** Demographics and clinical features before and after admission in patient hospitalized with 2009 H1N1 infection.

Characteristics Demographics	N	%
Age		
0 to 5 years old	41/117	41.9
6 to 20 years old	18/117	15.4
21 to 60 years old	35/117	29.9
> 60 years old	15/117	12.8
Gender. Female	60/117	51.3

**Clinical Features Before Admission**		

Fever >38.5 ºC	85/117	72.6
Dry Cough	81/117	69.2
Cough with sputum	16/117	13.7
Myalgia	11/115	9.6
Headache	3/117	2.6
Rhinorrhea	21/117	21.4
Low grade of fever	7/109	6.4
Cyanosis	16/116	13.7
Dyspnea	40/116	34.5
Chest pain	9/116	7.8
Vomiting	40/117	34.2
Diarrhea	45/116	38.8

**Clinical Features After Admission**		

Fever documented after admission	98/117	83.8
Pulse>100 beats per minute	96/115	83.5
Oxygen saturation values from pulse oximetry<95%	65/117	55.6
Cyanosis	19/115	16.5
Congested throat	56/117	47.9
Lethargy	25/117	21.4
Crackle	57/116	49.1
Wheezing	10/117	17.1
Respiratory rates >20 breaths per minute	82/84	97.6
Respiratory distress	49/117	42.2

**Table 2:** Co-morbidities in patient hospitalized with 2009 H1N1 infection.

Comorbidity	N	%
Kaposi Sarcoma	2/116	1.7
Type I Diabetes Mellitus	1/116	0.9
Pregnant women	10/95	10.5
Chronic Kidney Disease	5/116	4.3
Sickle cell anemia	8/116	6.9
Congenital cardiovascular disease	5/116	4.3
Asthma	7/116	6.8
Type II Diabetes Mellitus	4/103	3.9
Epilepsy	11/116	9.5
Hepatitis B	1/116	0.9
Immunosuppressive drugs	2/104	1.9

**Table 3:** Pertinent investigations in patient hospitalized with 2009 H1N1 infection.

Investigation	N	%age
Left sided infiltrates on chest x-ray	9/117	7.7
Right sided infiltrates on chest x-ray	24/117	20.5
Diffuse multilobar infiltrates on chest x-ray	9/117	7.7
Scattered bilateral infiltrates on chest x-ray	15/117	12.8
Normal chest x-ray	48/117	41.0
Hypoxia on arterial blood gases	47/83	56.6
Type II respiratory failure on arterial blood gases	7/83	8.5
Normal arterial blood gases	29/83	34.9
Hemoglobin (Anemia: Adult Male <14 gm/dl and Adult Female <13 gm/dl; children <11 gm/dl)	36/117	30.8
Platelets < 140 × 109 /ul	18/117	15.4
Platelets 140 to 400 × 109/ul	87/117	74.3
Platelets > 400 × 109/ul	12/117	10.3
White Blood Cell Count < 4 × 109/ul	11/117	9.4
White Blood Cell Count 4 to 11 × 109/ul	82/117	70.1
White Blood Cell Count > 11 × 109/ul	24/117	20.5
Total Bilirubin 3 to 17 umol/L	69/108	63.9
Total Bilirubin > 17 umol/L	39/108	36.1
Alanine transaminase (ALT)< 30 U/L	51/113	45.1
Alanine transaminase (ALT) 30 to 65 U/L	43/113	38.1
Alanine transaminase (ALT) > 65 U/L	19/113	16.8
Creatine kinase (CK) 21 to 232 U/L	65/111	58.6
Creatine kinase (CK) > 232 U/L	46/111	41.4
Lactate dehydrogenase (LDH) 100 to 500 U/L	105/117	89.7
Lactate dehydrogenase (LDH) > 500 U/L	12/117	10.3
Urea 2.5 to 7.5 mmol/L	79/117	67.5
Urea > 7.5 mmol/L	25/117	21.4

**Table 4:** Complications in patient hospitalized with 2009 H1N1 infection.

Complication	N	%age
CPAP*	2/117	1.7
CPAP then intubated	2/117	1.7
Intubated	16/117	13.7
None	97/117	82.9
Duration of stay in days; Median	4*	2–7 days; Median, (1^st^–3^rd^ Quantile)
Complications	20/104	19.2
Dead	18/108	16.7

* CPAP= Continuous positive airway pressure ventilation.
